# New World Hantavirus in Humans, French Guiana

**DOI:** 10.3201/eid1208.051619

**Published:** 2006-08

**Authors:** Séverine Matheus, Jean Baptiste Meynard, Pierre Rollin, Bertrand Maubert, Jacques Morvan

**Affiliations:** * Institut Pasteur de la Guyane, Cayenne, French Guiana;; † Centers for Disease Control and Prevention, Atlanta, Georgia, USA

**Keywords:** Hantavirus, Sin Nombre virus, seroprevalence, French Guiana, letter

To the Editor: Hantaviruses are etiologic agents for hemorrhagic fever with renal syndrome in Europe and Asia and for hantavirus pulmonary syndrome (HPS) in the Americas. These viruses belong to the family Bunyaviridae, genus Hantavirus. The natural reservoir of these viruses is wild or domestic rodents. HPS was first described in 1993 in the Four Corners region of the United States (*1*). It is a respiratory illness associated with the inhalation of aerosolized rodent excreta (urine and feces) contaminated with hantavirus particles. Sin Nombre virus (SNV) was the first etiologic agent of this syndrome. Since 1993, HPS has also been reported and confirmed in 6 countries in South America: Argentina, Bolivia, Brazil, Chile, Paraguay, Uruguay (*2**,**3*). Several distinct hantaviruses have been associated with HPS, including Juquituba virus in Brazil (*4*), Andes virus in Southern Argentina (*5*), and Laguna Negra virus in Paraguay (*6*).

French Guiana, an overseas French Administrative Unit in the Amazonian forest complex, is located on the northeastern coast of the South America between Brazil and Suriname. Ninety percent of its surface is tropical rain forest; the remaining 10% is a coastal plain, where 90% of the 200,000 inhabitants live. Cayenne and 2 adjacent towns, Remire and Matoury, constitute the main urban centers, with 80,000 inhabitants, ≈40% of the population. People live mainly in individual houses and small buildings. Many houses are built near forests, except those in the center of Cayenne. The outskirts of Remire and Matoury are surrounded by secondary rain forest, and those of Cayenne by wooded hills, where wild mammals such as rodents live in large numbers.

The prevalence of antibodies to New World hantavirus is unknown in French Guiana. Several cases of atypical pneumonia not linked to other etiologic agents (Coxiella burnetii, Histoplasma boydii), combined with identification of hantavirus rodent reservoirs in neighboring countries, prompted us to determine the seroprevalence of hantavirus in this area (*7**,**8*).

To estimate the prevalence of antibodies to New World hantavirus, we conducted a retrospective serologic survey of patients with symptoms compatible with HPS. Patients were from all areas of French Guiana: 64% from the urban centers, 7% from rural regions, and 30% from unspecified regions. From April 2002 through April 2004, a total of 420 serum samples were collected from patients with acute-phase febrile illness, unexplained acute respiratory syndrome, or bilateral interstitial pulmonary infiltrates. Diagnosis of Q fever was excluded by negative serologic results for immunoglobulin M (IgM), IgG, or both to C. burnetii (bioMérieux, Marcy-l'Etoile, France).

To detect patients with IgG antibodies to SNV, the ELISA described by Feldmann et al. was used (*9*). Briefly, an SNV-positive serum provided by the Centers for Disease Control and Prevention (CDC, Atlanta, GA, USA) was used as a positive control. Negative controls were obtained by random sampling of all previously negative samples. A sample was considered positive if the net absorbance values (after subtraction of absorbance values with and without antigen) were >0.2 for dilutions of 1:100 and 1:400 and the sum of 4 net absorbance values was >0.95. Seropositive samples were confirmed at CDC.

Antibodies reactive with SNV antigen indicate infection with a New World hantaviruses. However, because SNV is broadly cross-reactive with most New World hantavirus, the specific hantavirus cannot be identified.

The seroprevalence of IgG antibody to hantavirus was 1.42% (6/420) in the selected population. Three other samples showed borderline positivity. Antibody prevalence was not significantly different among the 7 age classes used (0–9, 10–19, 20–29, 30–39, 40–49, 50–59, and >60 years of age, p = 0.36, degrees of freedom = 6, by χ^2^ test) or by sex (p = 0.22, by Fisher exact test).

All patients with seropositive samples lived in the urban centers. The mean age of the 6 patients was 36.0 years (range 24–56 years), and 83% were men. Test results for IgM antibodies to SNV conducted on samples in parallel were negative.

The seroprevalence found in this study was caused by patient exposure to hantavirus. However, in the absence of IgM to SNV, we cannot link the respiratory symptoms observed to recent infection with hantavirus. Lack of information about the patients, especially their clinical history and details of travel to bordering countries, did not permit an association of infection with hantavirus contact in French Guiana. The seroprevalence observed is similar to that in Venezuela, where hantaviruses were isolated from rodents in 1999, but is lower than that observed in regions of Brazil (*10*).

The presence of hantaviruses in neighboring countries, as well as frequent travel by people in and out of French Guiana, has encouraged us to continue studying these viruses. We plan to conduct a study to systematically evaluate hantaviruses by serologic analysis and genomic amplification in persons with suggestive pathology. This study will be carried out in parallel with an investigation of rodent reservoirs of hantaviruses.
